# Maspin expression in prostate tumor elicits host anti-tumor immunity

**DOI:** 10.18632/oncotarget.2615

**Published:** 2014-10-21

**Authors:** Sijana H. Dzinic, Kang Chen, Archana Thakur, Alexander Kaplun, R. Daniel Bonfil, Xiaohua Li, Jason Liu, M. Margarida Bernardo, Allen Saliganan, Jessica B. Back, Hiroshi Yano, Dana L. Schalk, Elyse N. Tomaszewski, Ahmed S. Beydoun, Gregory Dyson, Adelina Mujagic, David Krass, Ivory Dean, Qing-Sheng Mi, Elisabeth Heath, Wael Sakr, Lawrence G. Lum, Shijie Sheng

**Affiliations:** ^1^ Department of Pathology, Wayne State University School of Medicine, Detroit, Michigan; ^2^ Tumor Biology and Microenvironment Program, Barbara Ann Karmanos Cancer Institute, Detroit, Michigan; ^3^ Department of Obstetrics and Gynecology, Wayne State University School of Medicine, Detroit, Michigan; ^4^ Department of Immunology and Microbiology, Wayne State University School of Medicine, Detroit, Michigan; ^5^ Department of Oncology, Wayne State University School of Medicine, Detroit, Michigan; ^6^ Department of Perinatology Research Branch, Eunice Kennedy Shriver National Institute of Child Health and Human Development, National Institutes of Health (NIH), Detroit, Michigan; ^7^ Mucosal Immunology Studies Team, National Institute of Allergy and Infectious Diseases, NIH, Bethesda, Maryland; ^8^ Department of Urology, Wayne State University School of Medicine, Detroit, Michigan; ^9^ Henry Ford Health Systems, Detroit, Michigan; ^10^ Department of Medicine, Wayne State University School of Medicine, Detroit, Michigan; ^11^ Molecular Therapeutics Program, Barbara Ann Karmanos Cancer Institute, Detroit, Michigan; ^12^ Current address: BIOBASE Corporation, Beverly, Massachusetts

**Keywords:** prostate tumor xenograft, tumorigenicity, flow cytometry, CD11b^+^Ly6G^high^ neutrophils, neutrophil maturation and chemotaxis, B-cell antibody response, ^51^Cr-release assay, antibody-dependent cellular cytotoxicity, lymphangiogenesis, intratumoral fibrosis, angiogenesis, leukocyte-filled lytic and necrotic centers

## Abstract

The goal of the current study is to examine the biological effects of epithelial-specific tumor suppressor maspin on tumor host immune response. Accumulated evidence demonstrates an anti-tumor effect of maspin on tumor growth, invasion and metastasis. The molecular mechanism underlying these biological functions of maspin is thought to be through histone deacetylase inhibition, key to the maintenance of differentiated epithelial phenotype. Since tumor-driven stromal reactivities co-evolve in tumor progression and metastasis, it is not surprising that maspin expression in tumor cells inhibits extracellular matrix degradation, increases fibrosis and blocks hypoxia-induced angiogenesis. Using the athymic nude mouse model capable of supporting the growth and progression of xenogeneic human prostate cancer cells, we further demonstrate that maspin expression in tumor cells elicits neutrophil- and B cells-dependent host tumor immunogenicity. Specifically, mice bearing maspin-expressing tumors exhibited increased systemic and intratumoral neutrophil maturation, activation and antibody-dependent cytotoxicity, and decreased peritumoral lymphangiogenesis. These results reveal a novel biological function of maspin in directing host immunity towards tumor elimination that helps explain the significant reduction of xenograft tumor incidence *in vivo* and the clinical correlation of maspin with better prognosis of several types of cancer. Taken together, our data raised the possibility for novel maspin-based cancer immunotherapies.

## INTRODUCTION

Maspin, an epithelial-specific member of the serine protease inhibitor (serpin) superfamily, was first discovered in 1994 as a tumor suppressor in breast cancer [1]. Since then, a large number of clinical studies have shown that maspin down-regulation correlates primarily with cancer progression at the step of tumor invasion [2-5], and maspin expression correlates with better prognosis and better overall patient survival [2, 6-8].

Consistent with clinical data, functional studies revealed tumor suppressive functions of maspin in a range of biological processes in tumor cells, including cell differentiation, apoptosis, and angiogenesis [9-14]. We recently showed that maspin expression in prostate carcinoma cells was sufficient to drive prostate tumor cells through a spectrum of temporally and spatially polarized cellular processes of re-differentiation [12]. Genes commonly regulated by maspin were a subset of histone deacetylase 1 (HDAC1) target genes that were closely associated with epithelial differentiation and transforming growth factor-β (TGF-β) signaling. In the same study, we demonstrated that maspin functions as a master regulator of the transcription program inducing specific and significant changes in protein expression patterns involved in apoptosis [12, 13]. Moreover, we have previously shown that maspin can specifically sensitize tumor cells to drug induced apoptosis *in vitro* [15]. Maspin has been shown to reduce tumor-derived vascular endothelial growth factor (VEGF) expression and angiogenesis [11, 16].

Maspin displays unique biochemical and biophysical properties that deviate significantly from classical inhibitory serpins. It only inhibits serine protease-like targets and is further regulated by its subcellular compartmentalization [10, 14, 17-20]. Although maspin does not have any specific subcellular localization sequence motif, it has been found to be nuclear, cytosolic, cell membrane-associated and secreted protein [20]. Therefore, the mode of tumor suppressive function of maspin and its molecular interactions may depend on its subcellular localization. For example, we demonstrated clinical and *in vitro* evidence that nuclear maspin acts an endogenous inhibitor of HDAC1 [17], one of the most promising therapeutic targets for cancer [21]. We and others have shown that nuclear maspin, in particular, predicts better overall patient survival [7, 18, 22-27], perhaps because of its interaction and inhibition of HDAC1. Earlier, we also showed that cell surface associated maspin inhibits the cell surface-associated zymogen form of urokinase type plasminogen (pro-uPA), contributing to the inhibition of cell detachment, cell motility, extracellular matrix remodeling and tumor invasion [10, 14]. Independently, the inverse correlation between maspin and uPA has been demonstrated as a significant feature in prostate cancer metastasis [28]. These findings collectively demonstrate that maspin is a multi-faceted suppressor of epithelial tumorigenesis and stromal responses.

However, the role of maspin in host anti-tumor immune responses has not been elucidated.

Here, we utilized the athymic nude mouse model capable of supporting the growth and progression of xenogeneic human prostate cancer cells to investigate the role of maspin in host anti-tumor immunity. This mouse model retains innate and humoral immunity and is suitable for testing the immunotherapeutic responses against human cancer cells [29]. We provide the first evidence that maspin expression in the prostate cancer xenograft elicits neutrophil- and B cells-dependent host immunity to promote tumor elimination. These findings are likely to open a new avenue for the development of novel maspin-based cancer immunotherapies.

## RESULTS

### Maspin expression results in reduced tumor incidence and proliferation

**Figure 1:**
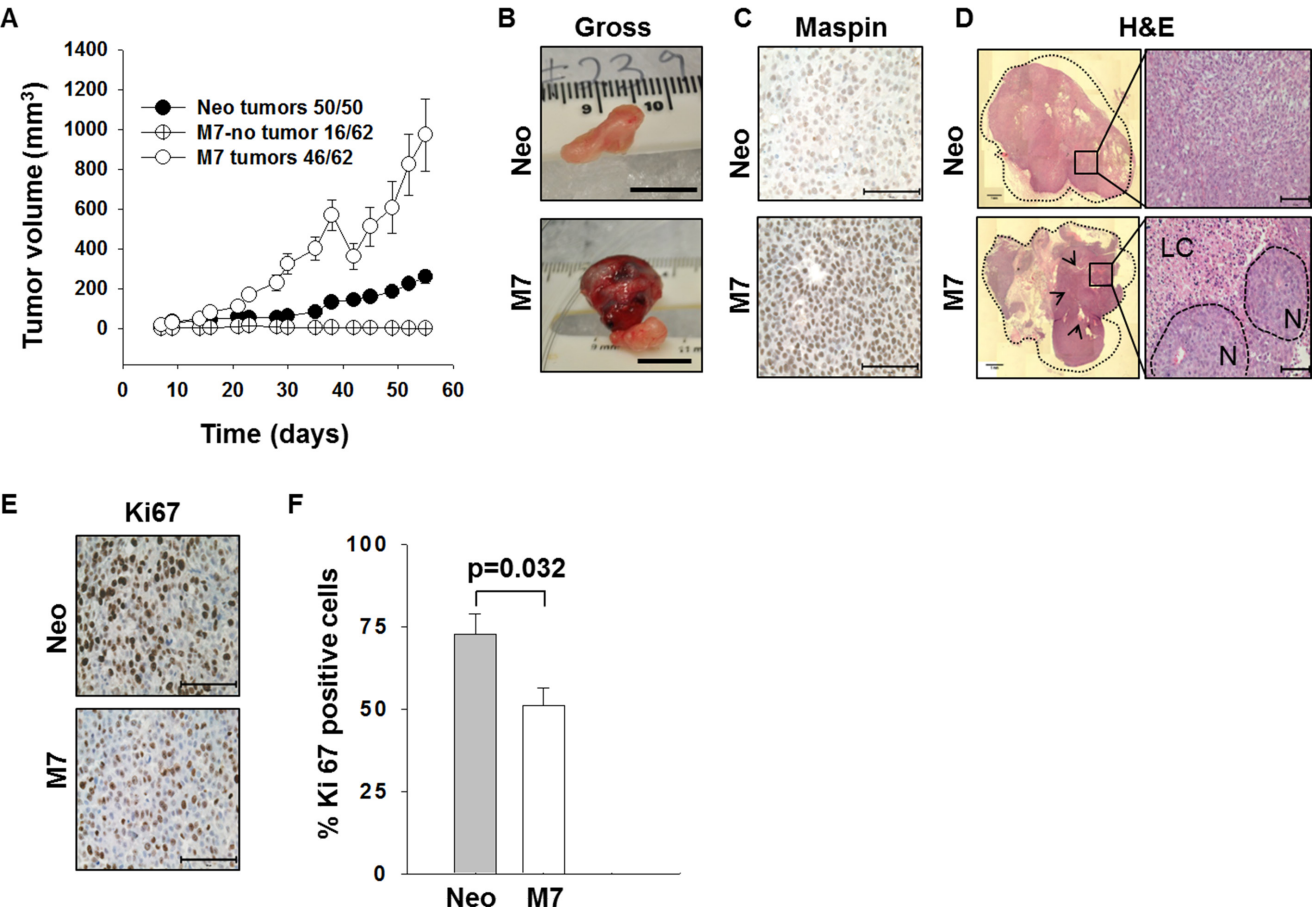
Characterization of prostate xenograft tumors A) Tumor growth curves. Each data point represents an average gross tumor volume ± standard error (SE). B) Representative gross tumor appearance (scale bar=1 cm). C) Representative IHC of maspin in xenograft tumors (scale bar=100 μm). D) Images of the entire tumor section (left, within the dashed outline) reconstructed from microscopic images of the H&E staining of (right, scale bar=100 μm). Black arrows in the left panel indicate the areas of tissue lysis. LC= lytic centers and N= epithelial-like nodules in the right panel, respectively. E) Representative microscopic images of the IHC of Ki67 (scale bar=100 μm). F) Quantification of proliferative cells based on IHC of Ki 67 (as shown in E), presented as an average percentage of Ki67^+^ cells in the total number of cells in each microscopic field. Three fields were counted for each tissue section (p=0.032). In C) and D), the corresponding antigen is stained brown, while the blue color is the nuclear counterstain.

To directly investigate the effect of maspin expression in tumor cells on tumor growth and interaction with the host environment *in vivo*, we inoculated athymic nude mice subcutaneously (s.c.) with either DU145 cells stably transfected with human maspin (M7) or those transfected with an empty vector (Neo). The animals bearing M7 or Neo tumors had similar body weights (Figure S1). The tumor incidence in mice inoculated with M7 cells was 74% (46/62), whereas 100% of mice inoculated with Neo cells developed tumors (50/50)*.* While the total volume of M7 tumors was larger than that of Neo tumors (*p* < 0.01) (Figure 1A), M7 tumors were found to contain a large volume of semi-solid fluid (Figure 1B). Consistently, M7 tumors exhibited a soft cyst-like texture and visible extravascular hemolysis. In contrast, Neo tumors were solidly packed with tumor cells without significant extravascular hemolysis. Histopathological examination of the lungs and regional lymph nodes showed no evidence of micro- or macro-metastasis in either Neo- or M7-tumor bearing mice (data not shown). Immunostaining confirmed low maspin expression in Neo tumors, in contrast to high maspin expression in M7 tumors (Figure 1C), demonstrating the stability of maspin transgene expression *in vivo*. Histological evaluation revealed a heterogeneous tissue composition of M7 tumors. These tumors were clustered in epithelial-like nodules surrounded by leukocyte-filled lytic and necrotic centers. In contrast, the homogeneous mass of poorly differentiated Neo cells showed no particular pattern of histological organization (Figure 1D). Furthermore, immunohistochemistry of proliferative marker Ki-67 showed that the M7 tumors were significantly less proliferative than the Neo tumors (Figures 1E and F, = 0.032). Therefore, the larger volume of M7 tumors was mainly due to the presence of a significant amount of fluid rather than increased tumor proliferation.

### Maspin expression promotes intratumoral fibrosis and lymphangiogenesis and inhibits angiogenesis

**Figure 2:**
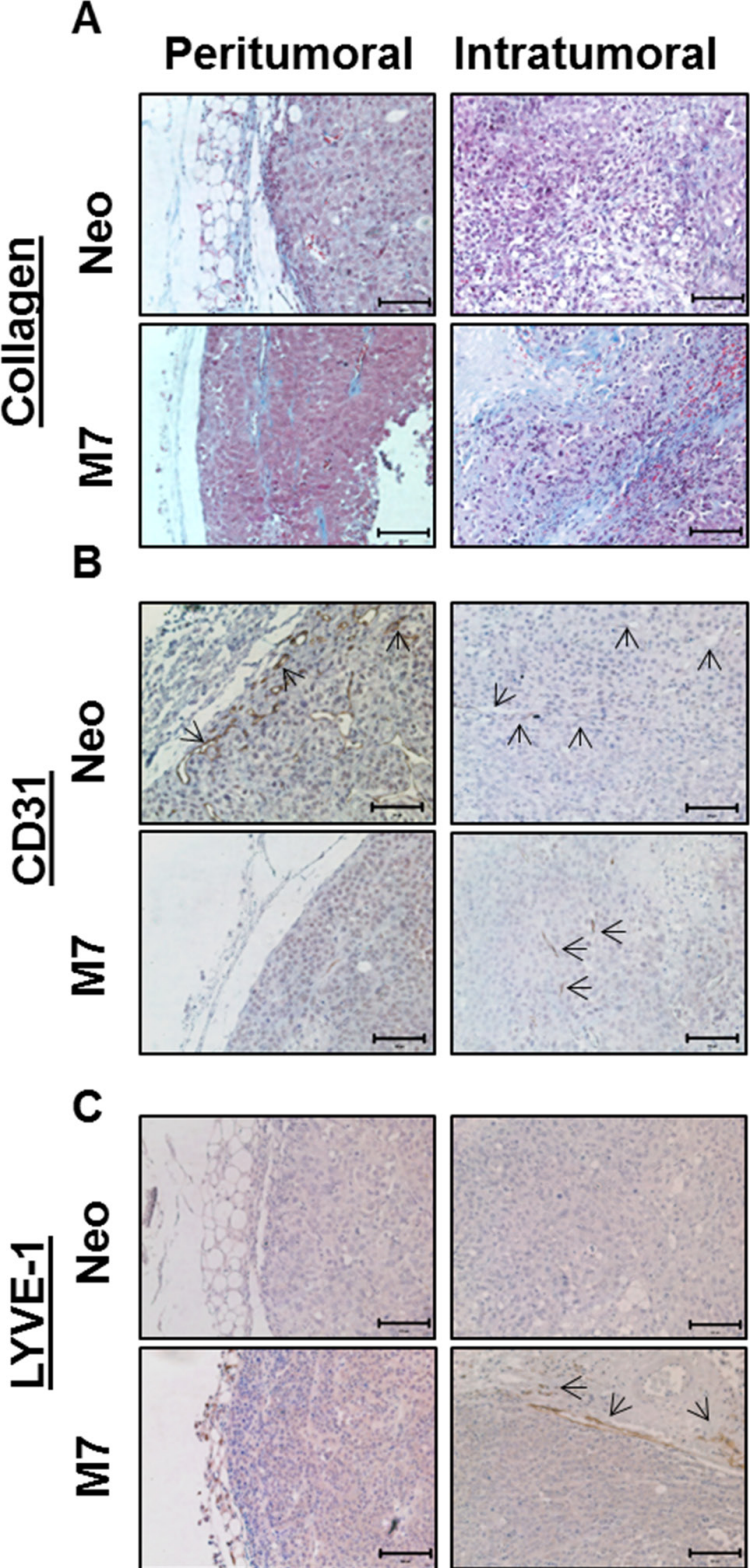
Characterization of maspin induced stromal reactivities A) Collagen staining (blue color) by Masson Trichrome staining .B) IHC of CD31 (brown color) and C) IHC of LYVE-1 (brown color), scale bar for all IHCs=100 μm. The arrows in B) point to the micro-blood vessels, whereas the arrows in C) point to the micro-lymphatic vessels.

To determine the function of maspin expression in the interaction between the tumor and the stroma, we performed Masson's Trichrome staining, and found elevated intratumoral fibrosis in M7 tumors (Figure 2A). While Neo tumors had increased fibrosis in the peritumoral area adjacent to the stroma and were almost devoid of collagen inside the tumors, M7 tumors were rich in intratumoral fibrosis. Immunostaining of CD31 showed that the Neo tumors were studded with micro blood vessels, whereas the M7 tumors exhibited only a few small blood vessels in the close proximity to the sites of leukocyte infiltration (Figure 2B). Interestingly, the marker of lymphatic endothelial cells, lymphatic vessel endothelial hyaluronan receptor-1 (LYVE-1), was not detected in the Neo tumors but was apparent at the interface between the solid and fibrotic masses of the M7 tumors and the stroma, suggesting increased interstitial pressure and lymphangiogenesis (Figure 2C). Thus, maspin expression in prostate tumor cells is associated with multifaceted inhibitory effects on tumor stromal reactivities.

### Maspin expression promotes neutrophil maturation and cytotoxicity in the tumor

**Figure 3:**
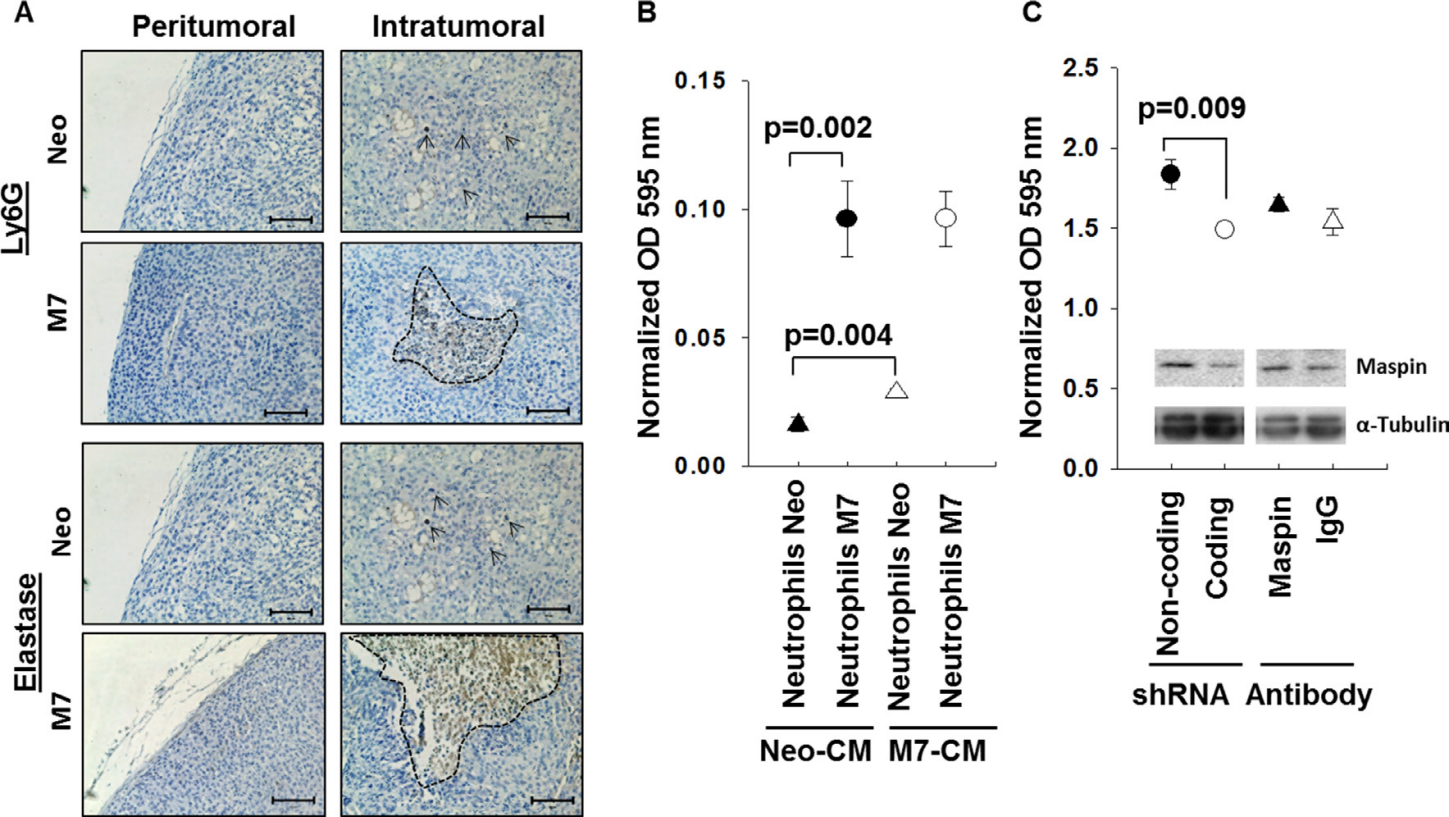
Characterization of neutrophil infiltration and motility A) IHC of Ly6G and neutrophil elastase in both the peritumoral and intratumoral tissue sections (scale bar=100 μm). The specific antigen is stained brown, whereas the nuclei are counterstained blue. Dashed lines mark neutrophil foci whereas the arrows point to individual neutrophil staining. B) Chemotaxis of splenic neutrophils from mice bearing either M7 or Neo tumors towards the CM of tumor cells in culture. The number of migrated cells, determined by the MTT assay, was quantified by spectrophotometric absorbance at 595 nm. The data were calibrated using the chemotaxis of the corresponding neutrophils to fresh serum free medium as a baseline. Data represent the average of three repeats, whereas the bars represent the standard deviation. C) Chemotaxis of neutrophils from naïve mice towards the CM of M7 cells with indicated treatments. The number of migrated cells, determined by the MTT assay, was quantified by spectrophotometric absorbance at 595 nm. The data were calibrated using the chemotaxis of the corresponding neutrophils to fresh serum free medium as a baseline. Data represent the average of three repeats, whereas the bars represent the standard deviation. The inset shows maspin expression after treatment. Alpha tubulin was used as a loading control.

Neutrophils are the first responders to be recruited to the site of tumor [30-33], and their activation is commonly associated with extracellular fibrosis [34]. We examined the Neo and M7 tumors for the neutrophil markers Ly6G and elastase. Neutrophils in Neo tumors were dispersed throughout the tumor tissues, whereas those in M7 tumors were organized and coincided with tumor necrotic foci (Figure 3A). Chemotaxis assay showed that splenic neutrophils from M7 tumor-bearing mice are more chemotactic in both Neo and M7 tumor cell-conditioned media (Figure 3B). In addition, M7 cell-conditioned media as compared to Neo, exhibited higher chemotactic properties to the neutrophils from Neo tumor-bearing mice. These data suggest that maspin-expressing tumors may induce neutrophil infiltration.

To determine whether intracellular maspin, secreted maspin or both are responsible for neutrophil recruitment, we silenced maspin expression in M7 cells by lentiviral shRNA or treated the M7 cells with a maspin-neutralizing antibody to block secreted maspin (Figure 3C). Silencing maspin expression in M7 cells, but not blocking secreted maspin, reduced neutrophil chemotaxis (p=0.009). These data suggest that the intracellular action of maspin is responsible for neutrophil migration.

**Figure 4:**
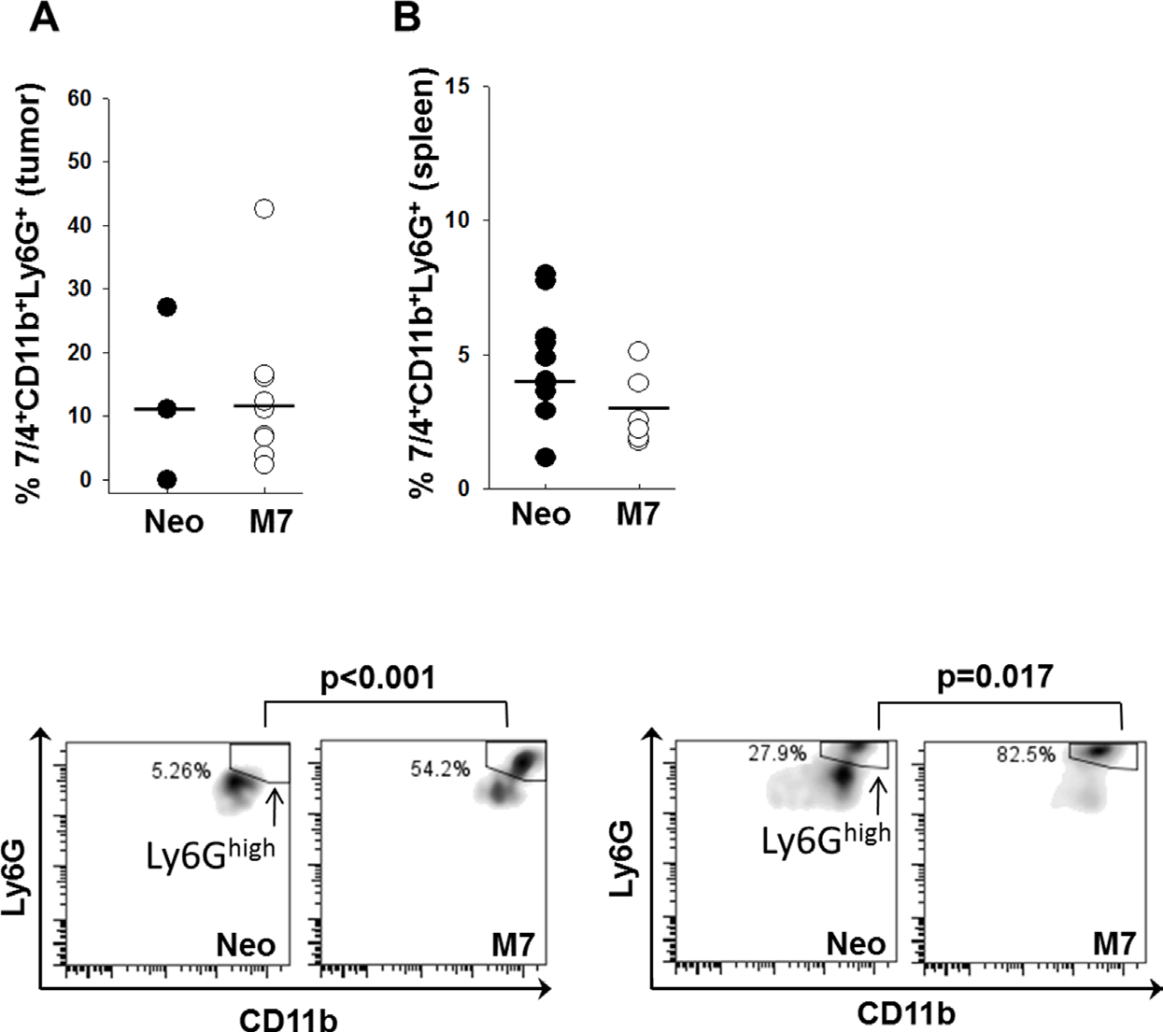
Characterization of neutrophils A) The quantification of 7/4^+^CD11b^+^Ly6G^+^tumor associated neutrophils (n=3 with Neo tumors; and n=11 with M7) and B) splenic neutrophils in tumor-bearing mice (n=10 with Neo tumors and n=6 with M7 tumors. C) and D) Representative histograms of 7/4^+^CD11b^+^Ly6G^high^ neutrophils in Neo and M7 xenograft tumors (p<0.001, based on multiple sample comparison) and in the splenocytes of tumor-bearing mice (p=0.017, based on multiple sample comparison). The horizontal bars represent the mean values.

**Figure 5:**
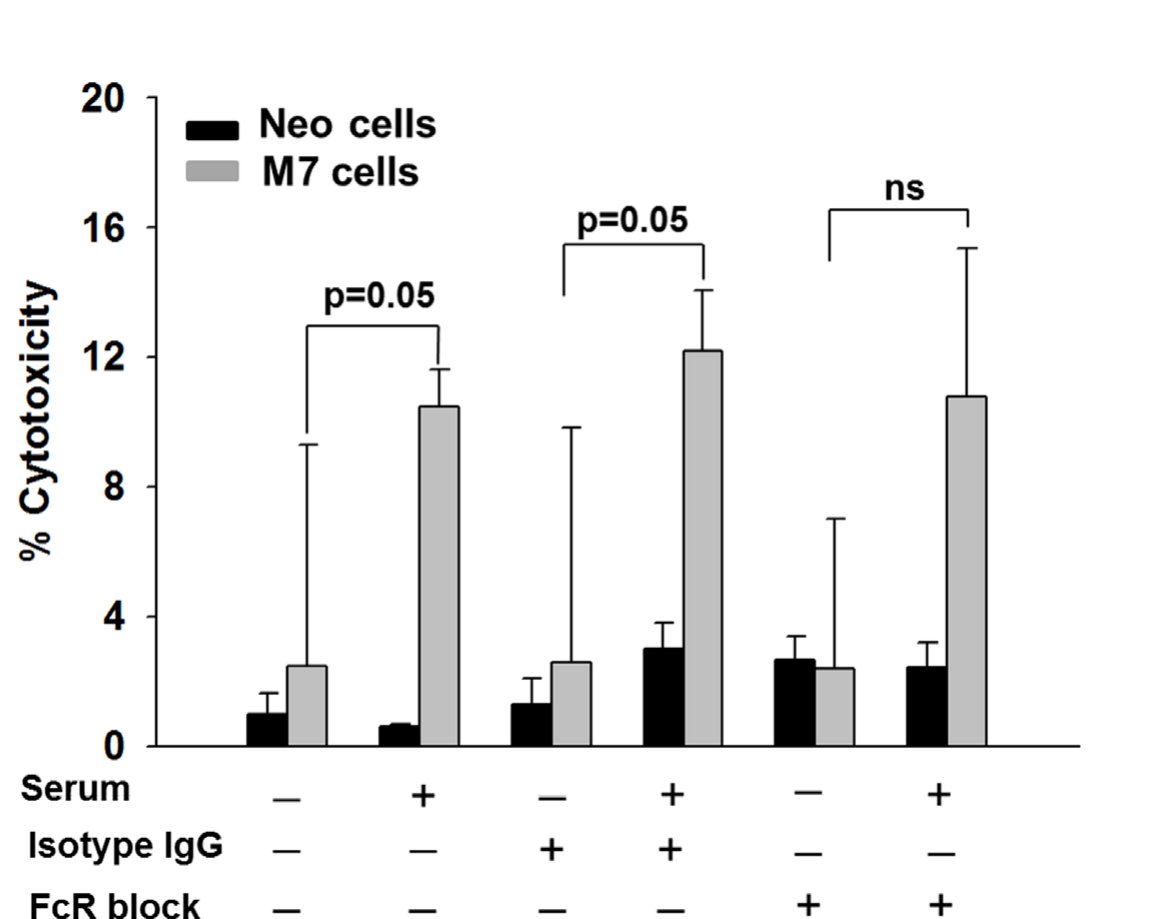
Cr-release assay Neutrophil-mediated cytotoxicity of tumor cells was measured by the ^51^Cr-release from labeled Neo or M7 cells and presented as the percentage of cytotoxicity in the presence or absence of serum, FcR CD16/CD32 Ab or isotype control, as specified. Data represent the average of three repeats, whereas the bars represent the standard errors (SE).

To determine the effect of maspin on the maturation and activation of neutrophils, we identified tumor-infiltrating and systemic neutrophils in tumor-bearing mice by flow cytometry. The gating strategy as illustrated in Figure S2. While Neo- and M7-tumoring bearing mice showed a similar percentage of 7/4^+^CD11b^+^Ly6G^+^ neutrophils in CD45^+^ leukocytes in the tumors and spleens (Figure 4A and B), there was a significantly higher proportion of Ly6G^high^ neutrophils in the tumors (p < 0.001) and the spleens (p = 0.017) of M7 tumor-bearing mice (Figure 4C and D). Neutrophils isolated from naive hosts and pre-stimulated with IFN-γ exhibited increased cytotoxicity towards maspin-expressing M7 cells (p = 0.05), which was further augmented upon the addition of the sera of tumor-bearing mice (Figure 5, = 0.05). Blockade of CD16 and CD32 abrogated serum-induced neutrophil cytotoxicity towards M7 cells, suggesting that neutrophil cytotoxicity towards M7 cells was partially mediated by antibodies in the sera of tumor-bearing mice. These results suggest that maspin-expressing tumors enhance the maturation and antibody-dependent activation of neutrophils in tumor-bearing hosts.

### Maspin-expressing tumors have increased antibody immunogenicity

**Figure 6:**
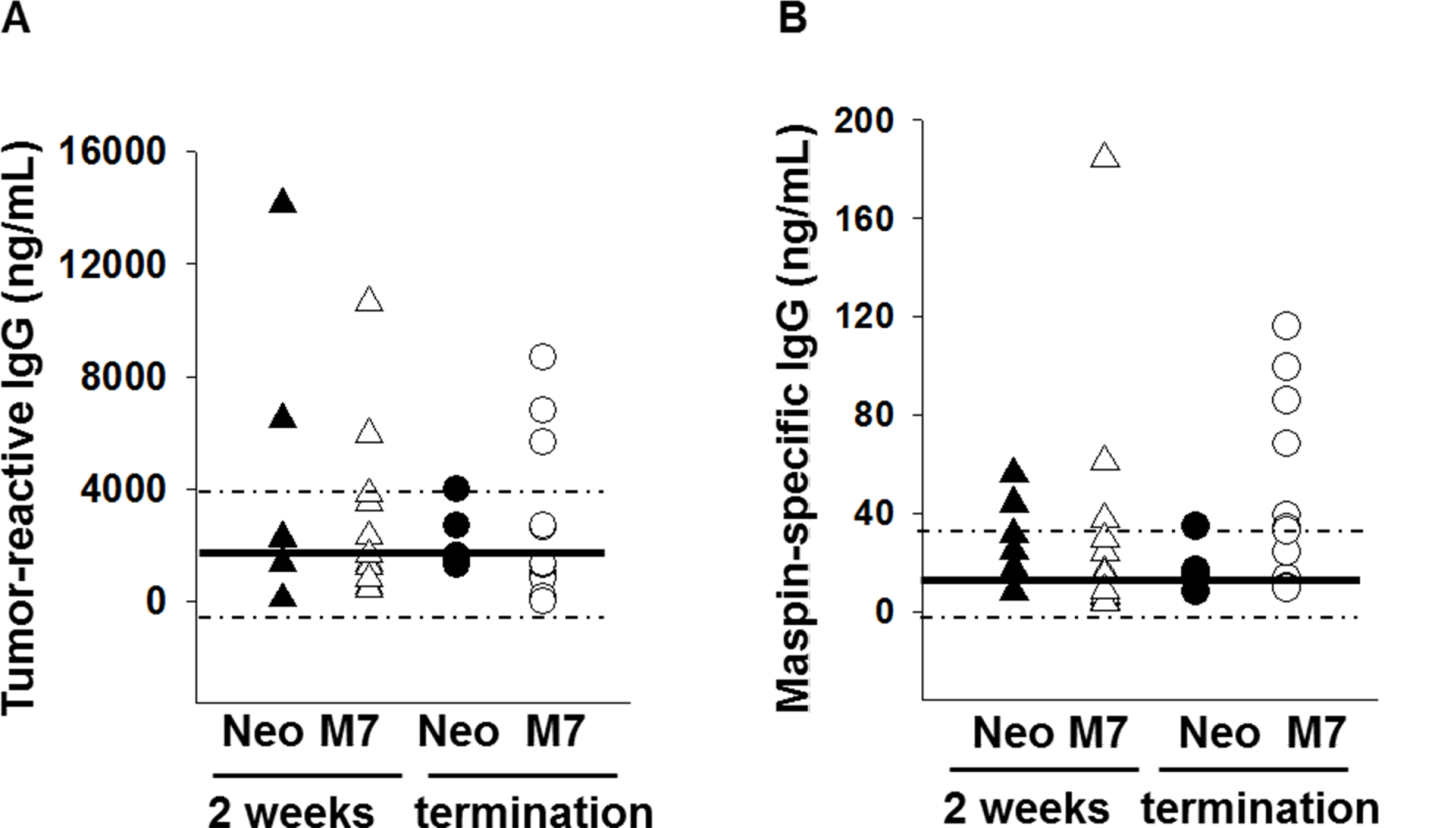
Quantification of tumor-reactive and maspin-specific IgG A) The quantification of tumor-responsive IgG and B) maspin specific IgG by ELISA in the sera of tumor bearing mice. The IgG was determined two weeks after the cells were inoculated (n=6 with Neo tumors and n=12 for M7 tumors) and at the termination point (n=5 with Neo tumors and n=12 with M7 tumors). The solid black line and the dashed lines represent the mean, the lowest and the highest IgG concentration, respectively, in the sera of control mice (n=5).

To determine if maspin expression in tumors results in enhanced tumor-specific antibody response that could stimulate neutrophil cytotoxicity, we analyzed the splenic B cell populations in Neo and M7 tumor bearing mice (Figure S2). No significant difference was noted in the percentage or composition of B cells in the spleen of Neo or M7 tumor-bearing mice (Figure S3A and B). However, increased levels of tumor cell-reactive and maspin-specific IgG was detected in the sera of a significant fraction of M7 tumor-bearing mice at the time of high tumor burden, which was not observed in Neo tumor-bearing mice (Figure 6A and B). Of note, this is the first biological evidence that maspin can function as a tumor antigen targeted by host antibodies. Collectively, these results demonstrate that maspin expression in the tumor enhances tumor-specific innate and antibody immune responses in the host.

## DISCUSSION

Accumulated clinical and *in vivo* evidence demonstrates that maspin is an intrinsic inhibitor of epithelial tumor metastasis. Metastasis is not an efficient process since tumor cells have to overcome a continuum of host anatomical and immunological barriers. The first *in vivo* evidence demonstrating tumor suppressive function of maspin utilized orthotropic breast cancer model in nude mice where it was shown that maspin expression in breast cancer cells lead to decreased tumor growth and metastasis compared to control [35]. Maspin overexpression in breast epithelial cells of C57Bl-6 WAP-TAg/WAP-maspin bi-transgenic mice was subsequently found to associate with increased apoptosis, decreased angiogenesis, and inhibition of tumor cell migration [36]. The functional role of maspin during the slow multi-stage breast tumor progression was investigated in a BALB/c MMTV/TGF-alpha transgenic mouse model where a direct correlation between maspin downregulation and tumor progression and metastasis was observed, and the loss of maspin expression paralleled the transition from carcinoma *in situ* to invasive carcinoma [37]. Using the SCID-Hu mouse model of prostate cancer bone metastasis, we showed that, in addition to inhibition of tumor growth, maspin expression in transfected prostate cancer DU145 cells induced glandular redifferentiation *in vivo* with concomitant reduction in bone osteolysis and angiogenesis and increase in fibrosis and collagen remodeling [11]. In addition, tumor metastasis mediated by nuclear cytokine-activated IKKα in the TRAMP model of prostate cancer could be suppressed by maspin [38].

In light of the significant reduction of tumor incidence in mice bearing maspin-expressing tumor cells, our findings demonstrate that, in addition to functioning as an intrinsic inhibitor of metastasis, maspin evokes tumor cell elimination by augmenting host immune surveillance. This is the first animal study to link the biological function of epithelial-specific tumor suppressor maspin to host anti-tumor immunity.

Neutrophils are the first innate immune effector cells recruited to wounds or tumor sites [30-33]. Consistent with this notion, we observed elevated recruitment and cytotoxicity of neutrophils in M7 tumors as compared to the Neo control. Furthermore, we demonstrated that intracellular maspin may play a crucial role in neutrophil recruitment. This result is underscored by our previous findings on nuclear maspin as an epigenetic regulator that controls the transcription of multiple genes encoding cytokines and chemokines important in neutrophil activation and migration such as IL-8, IL-24, CXCL10, TNFSF10 and TGF-β [12]. Consequently, the M7 tumors and the corresponding spleens harbored an elevated level of 7/4^+^CD11b^+^Ly6G^high^ cytotoxic neutrophils. It was demonstrated that tumor-associated neutrophils can exhibit two differentiation or activation states: an anti-tumorigenic “N1” state and a pro-tumorigenic “N2” state [39]. It is intriguing to speculate that N1 neutrophils in our animal model may be responsible for eliminating 26% of the M7 tumors.

Nonetheless, the need to have a T cell-deficient mouse model that supports human xenograft tumor growth limits our possibilities to study the full spectrum of anti-tumor immune response. However, considering that M7 and Neo cells did not elicit differential host immunity in T cell- and B cell-deficient SCID mice [11], the immune response observed in the B-cell competent athymic nude mice in the current study suggests an interplay between maspin-expressing tumors and host humoral immunity. Although B cell response is thought to be primarily dependent on CD4^+^ T cell help, recent studies showed that innate immune cells, including neutrophils, can promote the differentiation and activation of B cells independently of CD4^+^ T cells [40, 41]. Indeed, maspin-specific IgG in the sera of M7 tumor-bearing mice was elevated as compared to that from Neo tumor-bearing mice suggesting that surface-associated maspin or secreted maspin by M7 tumors may be as equally important in activating humoral immunity as intracellular maspin in activating neutrophils. To our knowledge, besides a report in a case of psoriasis, where maspin was reported to act as an autoantigen [42], this is the first study that demonstrates maspin-specific host immunity, thereby opening a new window of opportunity for maspin-based biomarkers and immunotherapy. Consistent with this notion, *in vitro* neutrophil cytotoxicity was enhanced by the sera of tumor-bearing mice. As a result of overall excessive tumor lysis and neutrophil infiltration, the significant accumulation of fluids in the M7 tumors might cause increased lymphangiogenesis, which further promotes leukocyte infiltration. Therefore, our finding on the augmentation of host innate and humoral immunity against tumors by maspin is relevant to the development of immunotherapeutic strategies for cancer patients with CD4^+^ T cell defects, such as AIDS patients.

Maspin is the only epithelial-specific endogenous HDAC inhibitor identified thus far [17]. Through a comprehensive comparative study, we previously showed that maspin reversed EMT by regulating a core group of HDAC-target genes closely associated with epithelial differentiation [12]. Furthermore, consistent with the maspin-associated transcriptome of epithelial homeostasis, M7 tumors featured less overall inflammatory stromal response, including less angiogenesis and more polarized collagen fibrosis which is a sign of partial completion of wound healing. Of particular importance, 8 out of 29 commonly downregulated genes, at the level of expression or activity, by maspin belong to the TGF-β pathway, specifically TGF-β, BMP5 and TGF-β receptor [12] Our data on the infiltration of cytotoxic neutrophils in M7 tumors, where TGF-β is downregulated, are consistent with the previous report showing that pharmacological blockade of TGF-β receptor blockade led to expansion and intratumoral infiltration of CD11b^+^Ly6G^+^cytotoxic neutrophils and their expression of pro-inflammatory cytokines [39].

It is important to note that extensive efforts have been devoted to targeting aberrant expression and activity of HDACs for cancer treatment [43-45]. While pharmacological HDAC inhibitors (HDACis) show promising efficacy in treating hematological malignancies, this strategy is not effective in treating solid tumors, at least in part due to their role in suppressing host immunity [46-48]. Taking into consideration that HDACs are ubiquitously expressed by all cell types and regulate many cellular processes, such as differentiation, proliferation, apoptosis and cellular immunity [49], systemic treatment with HDACis will inevitably cause adverse side effects, including further reducing host immune surveillance. An important lesson learned from the tumor-suppressive activities of maspin is that we can specifically target HDACs in tumor cells with maspin or maspin-mimetic compounds.

In this study we have utilized the animal model of prostate cancer to elucidate the role of maspin in host anti-tumor immune response. Of note, maspin expression is differentially regulated during prostate cancer and it is inversely correlated with tumor grade [50, 51]. On the other hand, the incidence of prostate cancer seems to correlate with chronic prostate inflammation [52, 53]. Furthermore, the activation of pro-tumor innate immunity seems to be positively correlated with the etiology of advanced prostate cancer [52]. Whether the loss of maspin expression during prostate cancer progression could play a causal role in the switch from anti-tumor to pro-tumor immunity remains to be determined with other prostate tumor cell lines in xenograft models and appropriate genetic mouse model for prostate cancer.

In conclusion, we show that maspin expression by tumor cells is capable of stimulating host anti-tumor innate and humoral immune responses. This discovery, coupled with our previous findings showing that maspin not only reprograms tumor cells for better differentiation but also increases tumor cell sensitivity to apoptosis [9, 11, 15, 54], may open a new window of opportunity to eradicate tumor with novel maspin-based tumor immunotherapy, alone or in combination with chemotherapy.

## MATERIAL AND METHODS

### Cell culture and reagents

Stable human maspin-transfected (M7) or mock-transfected control (Neo) cells, derived from the human prostate carcinoma cell line DU145 (American Type Culture Collection, Manassas, VA), were cultured as previously described [14]. The reagents and kits used in this study include: hematoxylin, eosin, and accustain Masson's trichrome (Sigma-Aldrich, St. Louis, MO); TMB (3,3′,5,5′-tetramethylbenzidine) substrate kit and DiffQuick staining kit (Fisher Scientific, Pittsburgh, PA); citrate buffer pH 6 (Life Technologies, Grand Island, NY), vectastain ABC kit (Vector Labs, Burlingame, CA), MTT (3-(4,5-dimethylthiazol-2-yl)-2,5-diphenyl tetrazolium bromide) assay kit (Millipore, Billerica, MA) and mono-poly resolving medium (MP Biomedical, Santa Ana, CA).

### Human prostate cancer xenograft model

Seven weeks old male athymic nude mice (Harlan Laboratories, Madison, WI) were inoculated subcutaneously (s.c.) with 2×10^6^ cells into the left flank. Tumor volume was measured twice a week with a caliper and calculated using the formula: A×B^2^/2, where A is the length of the tumor and B is the width of the tumor. The animals were divided into two groups. The first group was sacrificed at 2 weeks after inoculation, whereas the second group was sacrificed when the tumor volume reached 1cm^3^. During the experiments, mice were housed at the Division of Laboratory Animal Resources of Wayne State University. The animal protocol was approved by Institutional Animal Care and Use Committee in compliance with the animal welfare guidelines.

### Histopathological and molecular analysis

Xenograft tumors and mouse lungs were fixed in 4% paraformaldehyde, dehydrated with serial dilutions of ethanol and xylene, and embedded in paraffin. Five micrometer tissues sections were mounted on positively charged slides and dried for 1 hour at 60°C for histological and immunological analyses. The tissue sections were then deparaffinized and hydrated with xylene and serial dilutions of ethanol and rinsed in distilled water. For histopathological evaluation, hematoxylin and eosin (H&E) staining was performed as previously described [11]. To detect collagen fibrils, Masson's Trichrome Staining was performed according to the manufacturer's instructions. For immunohistochemical (IHC) staining, we adopted the procedure by Schwartz *et al* [55] using the specific primary and secondary antibodies (Abs) at the optimized dilutions, as summarized in Supplemental Table 1. As a negative control the primary Ab was replaced by isotypic preimmune IgG. The vectastain ABC kit was used for chromogenic detection of the bound horse radish peroxidase (HRP)-conjugated secondary Ab. All slides were evaluated under bright light using a DM IRB Leica microscope (Deerfield, IL).

### Flow cytometry

Splenocytes from tumor bearing mice were stained as previously described [56] using the Abs listed in Supplemental Table 1. Cells from the tumor tissues were homogenized using a Dounce homogenizer and were similarly stained. The fixable viability dye eFluor-450 was used to exclude non-viable cells from the analysis, and samples were fixed with 1% formaldehyde prior to analysis using a BD LSR II flow cytometer (BD Biosciences, San Jose, CA). Flow cytometry was performed in the Microscopy, Imaging, and Cytometry Resources (MICR) core at the Karmanos Cancer Institute and Wayne State University. Data were analyzed using FlowJo software (Tree Star, Ashland, OR).

### Enzyme-linked immunosorbent assay (ELISA)

ELISA for detecting tumor-reactive IgG was performed as previously described [57]. The amount of cell-bound IgG was calculated based on the standard curve generated with known amounts of human IgG in parallel. To detect maspin-specific IgG in mouse sera, flat bottom 96-well plates were coated with 1 ng/mL of recombinant maspin and incubated at 4 ºC overnight. The amount of maspin-specific IgG was calculated using the standard curve generated with known concentrations of maspin-specific Ab against recombinant maspin.

### Isolation and characterization of neutrophils from mouse blood and spleens

Fresh and pooled mouse blood, obtained by cardiac puncture, was laid over mono-poly resolving medium in 15 mL conical tubes and centrifuged at 300 g for 30 min at room temperature, to enrich neutrophils. The same resolving medium and method was used to enrich neutrophils in fresh splenocytes of tumor-bearing mice.

### Neutrophil chemotaxis assay

Tumor cells were cultured in serum free (SF) medium to 70% confluency. The M7 cells were also treated for 24h with either 5 μg/ml of maspin Ab or IgG control. To transiently silence maspin expression in M7 cells, the cells were transduced (48h) with lentivirus encoding maspin-specific or control shRNA as previously described [12]. 60 μL aliquots of Neo or M7-derived SF conditioned medium (SFCM) were placed in the lower compartment of 24-transwell plates. Fresh serum-containing medium was used as a negative control, whereas increasing concentrations of IL-8 (0.25, 0.50 and 1 ng/mL), added to the fresh maintenance medium, were used as positive control. 5×10^5^ splenic neutrophils/200 μL/well in serum-free medium from either tumor-bearing or naïve mice were added to the upper compartment of the transwells. The plate was incubated for 4 h at 37°C and the number of migrating cells in the lower compartments was determined using the MTT assay. Absorbance at 595 nm was measured using a microplate reader (Bio-Rad, Hercules, CA).

### ^51^Cr release assay

The M7 or Neo cells were seeded (1×10^4^ cells/200 μL/well in serum containing medium) in a 96-well plate and allowed to adhere overnight at 37°C. The next day, the cells were radiolabeled in maintenance media containing ^51^Cr at 20 μCi/mL for 6 hours at 37°C, washed 3 times and resuspended in culture medium. Naïve neutrophils were isolated from whole blood from 6-weeks old C57BL6/J mice. All neutrophils were pre-stimulated for 90 minutes in culture media with 10 ng/mL of IFN-γ. Some fractions of neutrophils were also pre-treated with the indicated Ab for an additional 45 min. The neutrophils were added to the ^51^Cr-labeled tumor cells at a ratio of 50 neutrophils to 1 tumor cell. Sera from tumor-bearing mice were added at 10% of the final volume. ^51^Cr-labeled tumor cells and neutrophils were co-cultured for 18 hours at 37°C. ^51^Cr released into the culture supernatants was measured using the Microbeta Trilux 1450 liquid scintillation counter (PerkinElmer, Waltham, MA). In parallel, the maximal ^51^Cr release was determined by adding 2% (v/v) SDS to ^51^Cr-labeled tumor cells without neutrophils. Spontaneous ^51^Cr release was determined in the presence of the maintenance medium only. Percent cytotoxicity was calculated as $\frac{\text{Experimental lysis}-\text{Spontaneous lysis}}{\text{Maximum lysis}-\text{Spontaneous lysis}}\times 100$

### Statistical analysis

Statistical significance was determined using either the Student's *t*-test (normally distributed data) or the Mann-Whitney U test (for data that failed normal distribution) as indicated. Differences were deems significance for *p* values less than 0.05.

## SUPPLEMENTARY METHODS, FIGURES AND TABLES


