# Supplementary material for: Maspin expression in prostate tumor elicits host anti-tumor immunity
Source: Oncotarget. 2014 Oct 21;5(22):11225–36. doi: 10.18632/oncotarget.2615 (PMC4294340; doi:10.18632/oncotarget.2615)
Supplement: Supplementary file 1 [file oncotarget-05-11225-s001.pdf]

Maspin expression in prostate tumor elicits host anti-tumor immunity

Supplementary Material

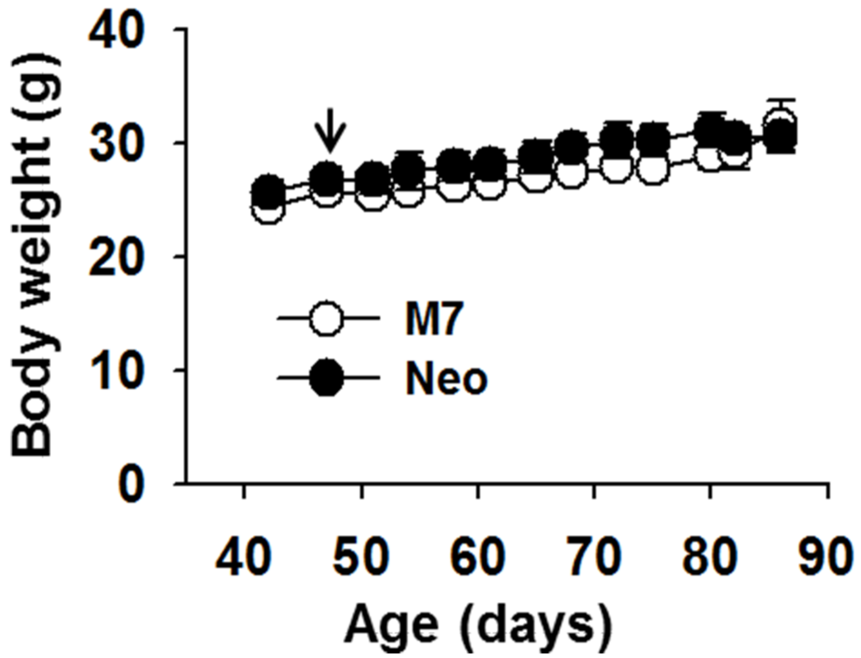

Supplemental Figure 1: Body weight of tumor-bearing mice.

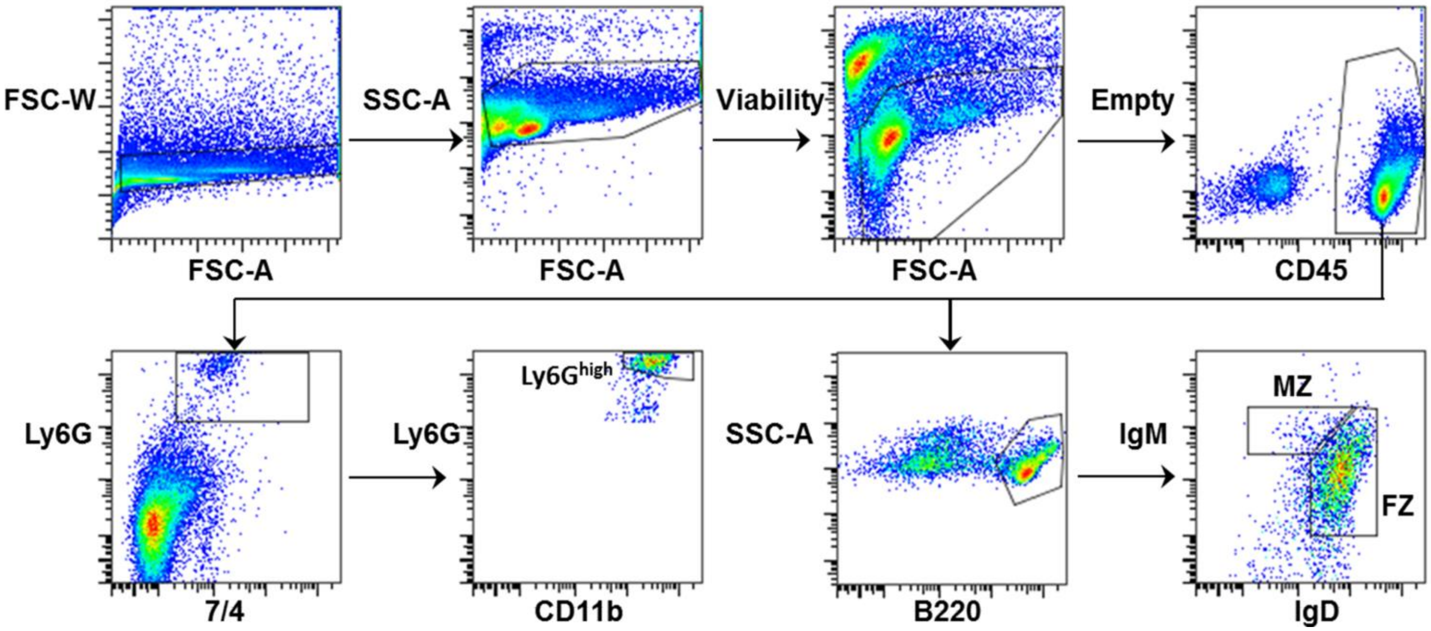

Supplemental Figure 2: Gating strategy for flow cytometry.

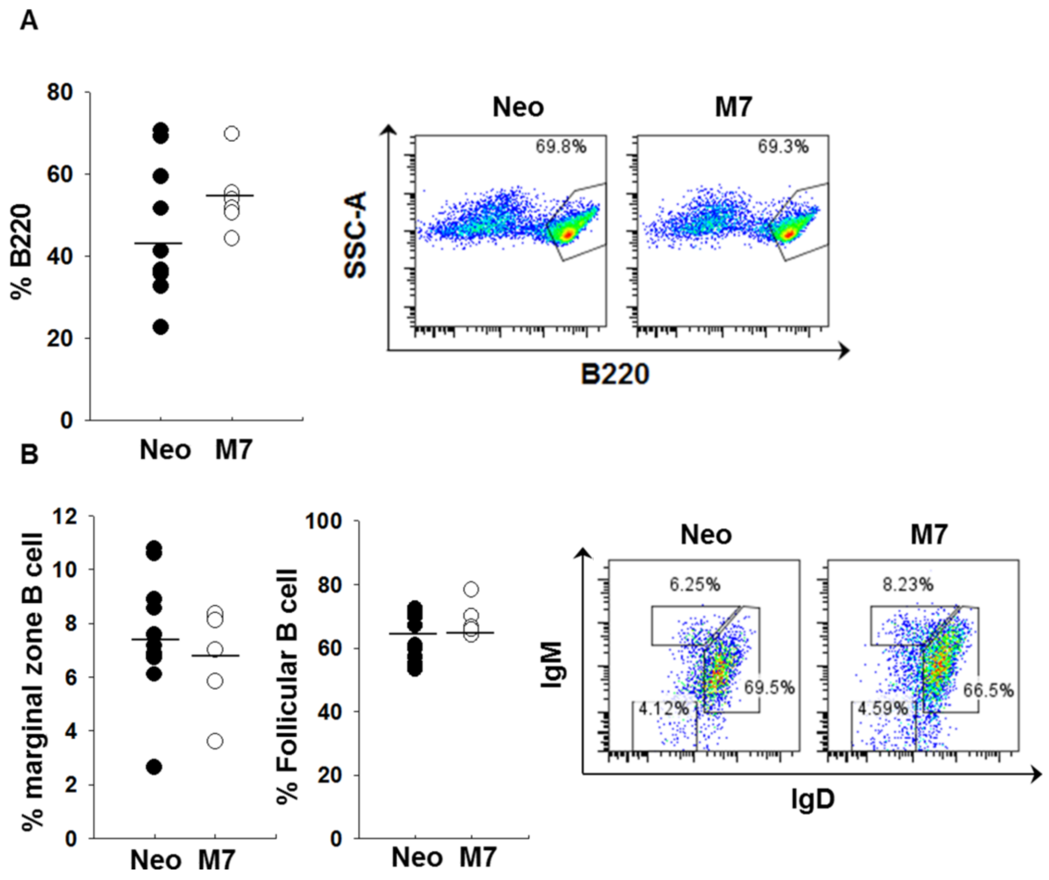

Supplemental Figure 3: Characterization of B cells.

Supplementary Table 1. The list of antibodies, sera and dyes			
**Antibody/Serum/Dye**	**Manufacturer**	**Catalogue number**	**Working Dilution**
CD31	Abcam	ab28364	1:500
Lyve1	Abcam	ab14917	1:1000
normal goat serum	Abcam	ab7481	
maspin	BD Pharmingen	554292	1:100
Elastase	Dako	M0752	1:1000
IgG1Ab	Millipore	MABC002	
Ki67	Vector Labs	VP-K452	1:200
biotinylated anti-mouse IgG	Vector Labs	BA-2000	
biotinylated anti-rabbit IgG	Vector Labs	BA-1000	
CD16/CD32	BD Biosciences	553141	10 ng/mL
IgG2b	BD Biosciences	553986	10 ng/mL
7/4 FITC	Abcam	ab53453	
IgM APC	BD Biosciences	550676	
IgD V450	BD Biosciences	560869	
CD45 PerCP-Cy5.5	e-Biosciences	45-0451-82	
CD11b APC	e-Biosciences	17-0112-82	
CD45R (B220) PE	e-Biosciences	12-0452-82	
fixable viability dye eFluor® 450	e-Biosciences	65-0863-18	
